# Remnant cholesterol is independently associated with diabetes, even if the traditional lipid is at the appropriate level: A report from the REACTION study

**DOI:** 10.1111/1753-0407.13362

**Published:** 2023-02-05

**Authors:** Binqi Li, Xin Zhou, Weiqing Wang, Zhengnan Gao, Li Yan, Guijun Qin, Xulei Tang, Qin Wan, Lulu Chen, Zuojie Luo, Guang Ning, Yiming Mu

**Affiliations:** ^1^ School of Medicine Nankai University Tianjin China; ^2^ Department of Endocrinology The First Medical Center of Chinese PLA General Hospital Beijing China; ^3^ Graduate School Chinese PLA General Hospital Beijing China; ^4^ Department of Medical Oncology The Fifth Medical Center of Chinese PLA General Hospital Beijing China; ^5^ The Second Medical Center of Chinese PLA General Hospital Beijing China; ^6^ Department of Endocrinology Ruijin Hospital, Shanghai Jiao Tong University School of Medicine Shanghai China; ^7^ Department of Endocrinology Dalian Central Hospital Dalian China; ^8^ Department of Endocrinology Zhongshan University Sun Yat‐sen Memorial Hospital Guangzhou China; ^9^ First Affiliated Hospital of Zhengzhou University Zhengzhou China; ^10^ Department of Endocrinology First Hospital of Lanzhou University Lanzhou China; ^11^ Department of Endocrinology Southwest Medical University Affiliated Hospital Luzhou China; ^12^ Wuhan Union Hospital, Huazhong University of Science and Technology Wuhan China; ^13^ First Affiliated Hospital of Guangxi Medical University Nanning China

**Keywords:** atherosclerosis, diabetes, dyslipidemia, remnant cholesterol, 残留胆固醇, 糖尿病, 血脂异常, 动脉粥样硬化。

## Abstract

**Background:**

The association between remnant cholesterol (RC) and diabetes remains unclear because of limited study and data. This study attempted to explore the association between RC and diabetes in a large sample, multicenter general population.

**Methods:**

The current study included 36 684 participants from eight provinces across China. Subjects were quartered according to the RC quartile. Logistic regression analysis was used to evaluate the association between RC and diabetes.

**Results:**

After adjusting for potential confounding factors, RC was still significantly associated with diabetes (Q4: odds ratio [OR]:1.147, 95% confidence interval [CI]: 1.049–1.254, *p* = .003). In addition, RC and diabetes were still significantly associated when triglycerides (TG) were <1.7 mmol/L (Q4: OR: 1.155, 95% CI: 1.005–1.327, *p* = .042), low‐density lipoprotein cholesterol (LDL‐C) <3.4 mmol/L (Q4: OR: 1.130, 95% CI: 1.011–1.264, *p* = .032), or HDL‐C (high‐density lipoprotein cholesterol) ≥1.0 mmol/L (Q4: OR: 1.116, 95% CI: 1.007–1.237, *p* = .037). In the stratification analysis, elevated RC was significantly associated with diabetes in subjects with systolic blood pressure (SBP) <140 mm Hg and diastolic blood pressure (DBP) <90 mm Hg, 60 ≤ estimated glomerular filtration rate (eGFR) ≤90 ml/min per 1.73 m^2^, younger than 55 years old and female.

**Conclusion:**

In the Chinese community, RC is significantly correlated with diabetes, even when TG, LDL‐C, or HDL‐C were controlled within the appropriate range recommended by the guidelines.

## Graphical abstract

**Highlights**

- The first multicenter, large‐sample study of the relationship between remnant cholesterol (RC) and diabetes.
- RC is independently associated with diabetes.
- RC represents residual diabetes risk beyond traditional lipids.

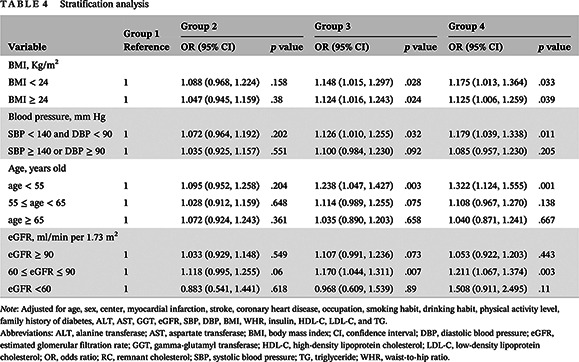

## 摘要

**背景:** 由于研究和数据的限制, 残留胆固醇(RC)与糖尿病之间的关联仍不清楚。本研究试图在中国大样本、多中心普通人群中探讨RC与糖尿病之间的关联。

**方法:** 本研究包括来自中国八个省份的36684名参与者。根据RC四分位数对受试者分组。采用 Logistic 回归分析评估RC和糖尿病之间的关联。

**结果:** 在校正潜在混杂因素后, RC仍然与糖尿病显著相关(Q4:1.147, 95% CI:1.049‐1.254, P=0.003)。此外, 在三酰甘油(TG)<1.7mmol/L, 低密度脂蛋白胆固醇(LDL‐C)<3.4mmol/L或高密度脂蛋白胆固醇(HDL‐C)≥1.0mmol/L的范围内, RC和糖尿病仍然显著相关(Q4: OR:1.155, 95% CI:1.005‐1.327, P=0.042；Q4: OR:1.130, 95% CI:1.011‐1.264, P=0.032；Q4: OR:1.116, 95% CI:1.007‐1.237, P=0.037)。分层分析中, 在收缩压<140 mmHg、舒张压<90 mmHg、60≤eGFR≤90 ml/min/ 1.73 m^2^、年龄在55岁以下的女性糖患者中, RC升高与糖尿病显著相关。

结论:在中国社区中, 即使在指南推荐的适当范围内控制TG、LDL‐C或HDL‐C, RC仍与糖尿病显著相关。

## 1 INTRODUCTION

Diabetes is one of the most serious epidemic diseases, resulting in a 2 ‐ to 3‐fold increasing danger of cardiovascular disease (CVD),^1^ a 2 ‐ to 3‐fold increasing danger of all‐cause mortality, and a 20‐year reduction in life expectancy.^2^, ^3^ As of 2021, 536.6 million people aged 21 to 79 worldwide were reported to have diabetes.^4^ The worrying thing is that if the prevalence of diabetes continues to rise dramatically, it is expected that by 2045, approximately 10.9% of the global population (about 700 million people) would have diabetes.^5^ However, current measures for preventing hyperglycemia are poorly beneficial, so there is an urgent need to find novel risk factors associated with diabetes.

In recent years, researchers have concentrated on the influence of dyslipidemia on diabetes.^6^ Previous studies have found that people who take lipid‐lowering drugs have a lower risk of diabetes.^7^, ^8^ Besides, a cohort study indicated that patients with dyslipidemia had 1.7 times the risk of diabetes than those with normal lipids.^9^ However, few studies have focused on the relationship between diabetes and remnant cholesterol (RC), which is a hot topic of research in the cardiovascular field in recent years and is considered an important cause of the residual risk of CVD.^10^

RC is the metabolic residues of triglyceride‐rich lipoproteins (TGRL). To be specific, it refers to the metabolic residues of very‐low‐density lipoprotein (VLDL), intermediate‐density lipoprotein (IDL), and chylomicron when not fasting and the metabolic residues of VLDL and IDL when fasting.^11^ It has been reported that the higher the level of RC, the greater the likelihood of developing CVD,^10^ especially in people with diabetes.^12^ Recent studies have also revealed a positive association between RC and diabetic complications.^13^, ^14^ However, large sample, multicenter studies on the relationship between RC and diabetes have not yet been reported. Hence, the purpose of the current study is to investigate the relationship between RC and diabetes in a large sample, multicenter Chinese community population.

## 2 METHODS

### 2.1 Study population

The data of the current work was from eight centers of the REACTION (Risk Evaluation of cAncers in Chinese diabeTic Individuals) study.^15^ A total of 53 639 participants aged over 40 or older participated in the study between March and December 2012. A total of 3042 participants with preexisting kidney disease and other serious illnesses, 2231 participants using lipid‐lowering drugs, 11 280 participants with missing important data, and 402 participants with calculated RC values ≤0 were excluded. Finally, 36 684 participants were enrolled.

### 2.2 Data collection

Trained investigators helped participants fill out detailed questionnaires including demographic data, past medical history, family history of diabetes, current medication, current smoking and drinking habits, current occupation, and physical activity. Information on physical activity was gathered using the short form of the International Physical Activity Questionnaire: high‐level physical activity was classified as vigorous physical activity that lasted longer than 10 min each within the past 7 days, which would make people breath with difficulty, for example, playing basketball, swimming, and running; moderate‐level physical activity was defined as a minimum of 10 min of physical activity in each of the past 7 days, which required people to breathe a little harder than normally, for instance jogging, playing table tennis, golf, or tai chi but no vigorous physical activity; low physical activity levels were determined as no physical activity or only some physical activity like walking, but could not meet the criteria for high‐ and medium‐level physical activity.

Systolic blood pressure (SBP) and diastolic blood pressure (DBP) were measured three times, each 5 min apart. The average of the three results was taken for statistical analysis. The height, weight, waist circumference (WC), and hip circumference were measured and recorded after the participants removed jackets and shoes.

The participants fasted for 8–10 h before the assay. Serum TG, low‐density lipoprotein cholesterol (LDL‐C), high‐density lipoprotein cholesterol (HDL‐C), total cholesterol (TC), aspartate transferase (AST), alanine transferase (ALT), glutamine transferase (GGT), serum creatinine (SCr), and other biochemical indexes were measured on an autoanalyzer; hemoglobin A1c (HbA1c) was measured by high‐performance liquid chromatography method; Fasting insulin was measured with chemiluminescent immunoassay. After fasting blood glucose extraction, participants without or with diabetes were tested for 75 g oral glucose tolerance or 100 g steamed‐bread meal, respectively, post‐load blood glucose (PBG) was extracted 2 h after the first blood sample was drawn.

### 2.3 Calculations

RC = TC−HDL‐C−LDL‐C.^16^ Body mass index (BMI) (kg/m^2^) = weight (Kg)/height^2^ (m). Waist‐to‐hip ratio (WHR) = WC/HC. eGFR (mL/min per 1.73 m^2^) = 175 × (SCr in mg/dL) −1.154 × age−0.203 × (0.742 for women) × (1.212 if African American).

### 2.4 Definitions

RC was divided into four groups based on quartiles: <25% group (the control group), 25%–50% group, 50%–75% group, and ≥75% group. Diabetes was defined according to the American Diabetes Association's criteria^17^: FPG ≥7.0 mmol/L or PBG ≥11.1 mmol/L or HbA1c ≥6.5% or self‐reported diabetes.

### 2.5 Statistical analysis

Statistical analysis was performed using SPSS 24.0 (IBM, Chicago, IL). Continuous variables were expressed as mean ± SD and categorical variables were presented numerically (proportionally). One‐way analysis of variance (ANOVA) was used to test the difference between continuous variables and the chi‐square test was used to analyze the categorical variables. The association between RC and diabetes was delineated by logistic regression analyses. Adjusted variables were examined using collinearity diagnosis according to the following criteria: (1) variance inflation factor >5; (2) condition index >30; and (3) variance proportions >50%. The adjusted variables had the following criteria: (1) in the current study, there was a significant difference between the diabetic group and the nondiabetic group, or it was significant for the occurrence and development of diabetes in clinical practice; and (2) there was no collinearity. Model 0 was unadjusted. Model 1 was adjusted for age, sex, and center. Model 2: Model 1 + myocardial infarction (MI), stroke, coronary heart disease (CHD), occupation, smoking habit, drinking habit, physical activity level, and family history of diabetes. Model 3 = Model 2 + ALT, AST, GGT, and eGFR. Model 4 = Model 3 + SBP, DBP, BMI, WHR, and insulin. Model 5 = Model 4 + HDL‐C and LDL‐C. Model 6 = Model 5 + TG. Moreover, according to the appropriate levels of LDL‐C, HDL‐C, and TG recommended by the 2016 edition of the guidelines for the management of dyslipidemia in Chinese adults,^18^ the association between RC and diabetes was also tested when LDL‐C <3.4 mmol/L, HDL‐C ≥1.0 mmol/L, and TG <1.7 mmol/L, respectively. In addition, the current study explored the relationship between RC and diabetes in BMI, BP, age, and eGFR subgroups. All statistical tests were two‐sided and *p* < .05 was considered statistically significant.

## 3 RESULTS

### 3.1 Clinical characteristics of the study population

The present study included 36 684 participants (Table 1), with 11 321 (30.9%) males and 25 363 (69.1%) females, and their median age (Q1–Q3) was 57 (52, 64). Compared with participants without diabetes, participants with diabetes had older age, higher RC, LDL‐C, TC, TG, BMI, WHR, ALT, AST, GGT, SBP, and DBP and lower HDL‐C and eGFR. Meanwhile, males, frequent smoking, MI, stroke, CHD, manual labor, retirement or unemployment, and a family history of diabetes were more common in participants with diabetes than in participants without diabetes.

**TABLE 1:** Characteristics of study population by diabetes

Variable	Total	No diabetes	Diabetes	*p* value
*N*	36 684	27 268	9416	
RC, mmol/L	0.69 (0.49, 0.95)	0.47 (0.66， 0.91)	0.55 (0.77, 1.08)	<.001
Age, years	57 (52, 64)	56 (51, 62)	60 (55, 68)	<.001
Sex, (%)				<.001
Male	11 321 (30.9)	7817 (28.7)	3504 (37.2)	
Female	25 363 (69.1)	19 451 (71.3)	5912 (62.8)	
LDL‐C, mmol/L	2.93 (2.36, 3.55)	2.93 (2.36, 3.53)	2.96 (2.36, 3.58)	.013
HDL‐C, mmol/L	1.29 (1.09, 1.52)	1.32 (1.11, 1.55)	1.21 (1.03, 1.42)	<.001
TC, mmol/L	5.05 (4.32, 5.80)	5.04 (4.32, 5.76)	5.10 (4.33, 5.89)	<.001
TG, mmol/L	1.37 (0.98, 1.97)	1.29 (0.93, 1.83)	1.65 (1.15, 2.36)	<.001
BMI, Kg/m^2^	24.27 (22.13, 26.57)	21.84 (23.92, 26.21)	25.20 (23.12, 27.53)	<.001
WHR	0.89 (0.84, 0.93)	0.88 (0.83, 0.92)	0.90 (0.86, 0.95)	<.001
ALT, U/L	15 (11, 21)	14 (10, 20)	16 (12, 24)	<.001
AST, U/L	20 (17, 25)	20 (17, 24)	21 (17, 26)	<.001
GGT, U/L	21 (15, 32)	19 (14, 29)	25 (17, 39)	<.001
SBP, mm Hg	129 (117, 144)	127 (115, 141)	136 (123, 150)	<.001
DBP, mm Hg	77 (70, 84)	76 (70, 83)	78 (71, 85)	<.001
eGFR, mL/(min·1.73 m^2^)	89.78 (80.09, 100.68)	90.43 (81.09, 00.94)	87.80 (77.27, 99.73)	<.001
Drinking, (%)				.664
Never	27 424 (74.8)	20 283 (74.4)	7141 (75.8)	
Occasional	6791 (18.5)	5230 (19.2)	1561 (16.6)	
Frequently	2467 (6.7)	1755 (6.4)	714 (7.6)	
Smoking, (%)				<.001
Never	31 477 (85.8)	23 520 (86.3)	7957 (84.5)	
Occasional	864 (2.4)	617 (2.3)	247 (2.6)	
Frequently	4343 (11.8)	3131 (11.5)	1212 (12.9)	
Physical activity, (%)				.09
Low	29 283 (79.9)	21 590 (79.2)	7693 (81.7)	
Moderate	5165 (14.1)	3971 (14.6)	1194 (12.7)	
High	2236 (6.1)	1707 (6.3)	529 (5.6)	
Occupation (%)				.011
Manual labor: worker/peasant/soldier	4399 (12.0)	3283 (12.0)	1116 (11.9)	
Administrative staff/doctor/teacher/scientist	1124 (3.1)	840 (3.1)	284 (3.0)	
Individual household/merchant	2728 (7.4)	2250 (8.3)	478 (5.1)	
Retire/unemployed	28 433 (77.5)	20 895 (76.7)	7538 (80.1)	
Myocardial infarction, (%)				<.001
Yes	123 (0.3)	62 (0.2)	61 (0.6)	
No	36 561 (99.7)	27 206 (99.8)	9355 (99.4)	
Stroke, (%)				<.001
Yes	425 (1.2)	265 (1.0)	160 (1.7)	
No	36 259 (98.8)	27 003 (99.0)	9256 (98.3)	
Coronary heart disease, (%)				<.001
Yes	1270 (3.5)	720 (2.6)	550 (5.8)	
No	35 414 (96.5)	26 548 (97.4)	8866 (94.2)	
Family history of diabetes, (%)				<.001
Yes	6428 (17.5)	4203 (15.4)	2225 (23.6)	
No	30 256 (82.5)	23 065 (84.6)	7191 (76.4)	

*Note*: Data were mean ± SD or median (interquartile range) for skewed variables or numbers (proportions) for categorical variables.

Abbreviations: ALT, alanine transferase; AST, aspartate transferase; BMI, body mass index; DBP, diastolic blood pressure; eGFR, estimated glomerular filtration rate; GGT, gamma‐glutamyl transferase; HDL‐C, high‐density lipoprotein cholesterol, LDL‐C, low‐density lipoprotein cholesterol; RC, remnant cholesterol; SBP, systolic blood pressure; TG, triglyceride; TC, total cholesterol; WHR, waist‐to‐hip ratio.

### 3.2 Association between RC and diabetes

Table 2 shows the association between RC and diabetes after potential confounders were adjusted and separated by sex. In the total participants, RC exhibited a significant adjusted odds ratio (OR) in models 0–6. After all potential confounders were adjusted in model 6, the third and fourth quartile of RC were still significantly associated with diabetes (Q3: OR: 1.132, 95% CI: 1.047–1.223, *p* = .002; Q4: OR:1.147, 95% CI: 1.049–1.254, *p* = .003). In the female participants, RC remained significantly associated with diabetes though all potential confounders were adjusted in model 6 (Q3: OR: 1.168, 95% CI: 1.057–1.291, *p* = .002; Q4: OR: 1.228, 95% CI: 1.097–1.975, *p* < .001). In the male participants, RC and diabetes were significantly associated in model 5 (Q3: OR: 1.133, 95% CI: 1.002–1.282, *p* = .046; Q4: OR: 1.144, 95% CI: 1.010–1.295, *p* = .034), however, after TG was adjusted in Model 6, the association between RC and diabetes no longer existed (Q4: OR: 0.985, 95% CI: 0.848–1.145, *p* = .846).

**TABLE 2:** Association between RC and diabetes in the total subjects, female subjects or male subjects

RC four categories	Model 0		Model 1		Model 2		Model 3		Model 4		Model 5		Model 6	
	OR (95% CI)	*p* value	OR (95% CI)	*p* value	OR (95% CI)	*p* value	OR (95% CI)	*p* value	OR (95% CI)	*p* value	OR (95% CI)	*p* value	OR (95% CI)	*p* value
Association between RC and diabetes in the total subjects														
Group 1	1		1		1		1		1		1		1	
Group 2	1.191 (1.110, 1.279)	<.001	1.116 (1.037, 1.201)	.003	1.105 (1.026, 1.189)	.008	1.093 (1.014, 1.179)	.02	1.091 (1.011, 1.178)	.026	1.088 (1.008, 1.175)	.031	1.063 (0.984, 1.148)	.119
Group 3	1.485 (1.385, 1.591)	<.001	1.355 (1.260, 1.456)	<.001	1.337 (1.243, 1.438)	<.001	1.266 (1.175, 1.364)	<.001	1.228 (1.138, 1.325)	<.001	1.196 (1.108, 1.291)	<.001	1.132 (1.047, 1.223)	.002
Group 4	2.145 (2.006, 2.294)	<.001	1.928 (1.798, 2.067)	<.001	1.893 (1.765, 2.031)	<.001	1.648 (1.532, 1.772)	<.001	1.477 (1.371, 1.592)	<.001	1.369 (1.269, 1.477)	<.001	1.147 (1.049, 1.254)	.003
Association between RC and diabetes in female subjects														
Group 1	1		1		1		1		1		1		1	
Group 2	1.253 (1.143, 1.374)	<.001	1.130 (1.028, 1.244)	.012	1.123 (1.020, 1.237)	.018	1.124 (1.019, 1.240)	.019	1.120 (1.013, 1.238)	.027	1.116 (1.010, 1.234)	.032	1.090 (0.986, 1.205)	.093
Group 3	1.655 (1.514, 1.809)	<.001	1.373 (1.252, 1.507)	<.001	1.358 (1.237, 1.491)	<.001	1.316 (1.196, 1.448)	<.001	1.279 (1.159, 1.411)	<.001	1.238 (1.122, 1.367)	<.001	1.168 (1.057, 1.291)	.002
Group 4	2.628 (2.413, 2.863)	<.001	2.050 (1.875, 2.242)	<.001	2.010 (1.836, 2.200)	<.001	1.827 (1.665, 2.005)	<.001	1.622 (1.474, 1.785)	<.001	1.479 (1.342, 1.631)	<.001	1.228 (1.097, 1.375)	<.001
Association between RC and diabetes in male subjects														
Group 1	1		1		1		1		1		1		1	
Group 2	1.161 (1.037, 1.300)	<.001	1.097 (0.978, 1.231)	.116	1.076 (0.957, 1.210)	.22	1.055 (0.937, 1.188)	.379	1.053 (0.934, 1.188)	.397	1.052 (0.933, 1.187)	.408	1.033 (0.915, 1.165)	.603
Group 3	1.347 (1.201, 1.511)	<.001	1.297 (1.154, 1.457)	<.001	1.272 (1.129, 1.431)	<.001	1.177 (1.043, 1.328)	.008	1.144 (1.012, 1.294)	.031	1.133 (1.002, 1.282)	.046	1.084 (0.956, 1.229)	.209
Group 4	1.577 (1.411, 1.763)	<.001	1.592 (1.421, 1.784)	<.001	1.558 (1.388, 1.749)	<.001	1.291 (1.144, 1.456)	<.001	1.196 (1.058, 1.352)	.004	1.144 (1.010, 1.295)	.034	0.985 (0.848, 1.145)	.846

*Note*: Model 0 was unadjusted. Model 1 was adjusted for age, sex, and center; model 2 was additionally adjusted for myocardial infarction, stroke, coronary heart disease, occupation, smoking habit, drinking habit, physical activity level, and family history of diabetes; Model 3 was additionally adjusted for ALT, AST, GGT, and eGFR; Model 4 was additionally adjusted for SBP, DBP, BMI, WHR, and insulin; Model 5 was additionally adjusted for HDL‐C and LDL‐C; Model 6 was adjusted for TG.

Abbreviations: ALT, alanine transferase; AST, aspartate transferase; BMI, body mass index; CI, confidence interval; DBP, diastolic blood pressure; eGFR, estimated glomerular filtration rate; GGT, gamma‐glutamyl transferase, HDL‐C, high‐density lipoprotein cholesterol; LDL‐C, low‐density lipoprotein cholesterol; OR, odds ratio; RC, remnant cholesterol; SBP, systolic blood pressure; TG, triglyceride; WHR, waist‐to‐hip ratio.

### 3.3 Association between RC and diabetes in participants with TG <1.7 mmol/L, LDL‐C < 3.4 mmol/L, or HDL‐C ≥1.0 mmol/L

In Table 3, RC and diabetes were still significantly associated when TG <1.7 mmol/L (Q4: OR: 1.155, 95% CI: 1.005–1.327, *p* = .042), LDL‐C <3.4 mmol/L (Q2: OR: 1.103, 95% CI: 1.008–1.208, *p* = .034; Q3: OR: 1.151, 95% CI: 1.047–1.264, *p* = .003; Q4: OR: 1.130, 95% CI: 1.011–1.264, *p* = .032), or HDL‐C ≥1.0 mmol/L (Q3: OR: 1.140, 95% CI: 1.045–1.243, *p* = .003; Q4: OR: 1.116, 95% CI: 1.007–1.237, *p* = .037).

**TABLE 3:** Association between RC and diabetes when TG <1.7, LDL‐C <3.4, HDL‐C ≥1.0 mmol/L

RC four categories	TG <1.7 mmol/L		LDL‐C <3.4 mmol/L		HDL‐C ≥1.0mml/L	
	OR (95% CI)	*p* value	OR (95% CI)	*p* value	OR (95% CI)	*p* value
Group 1	1		1		1	
Group 2	1.086 (1.000, 1.179)	.051	1.103 (1.008, 1.208)	.034	1.080 (0.991, 1.176)	.078
Group 3	1.063 (0.969, 1.166)	.198	1.151 (1.047, 1.264)	.003	1.140 (1.045, 1.243)	.003
Group 4	1.155 (1.005, 1.327)	.042	1.130 (1.011, 1.264)	.032	1.116 (1.007, 1.237)	.037

*Note*: Adjusted for age, sex, center, myocardial infarction, stroke, coronary heart disease, occupation, smoking habit, drinking habit, physical activity level, family history of diabetes, ALT, AST, GGT, eGFR, SBP, DBP, BMI, WHR, insulin, HDL‐C, LDL‐C, and TG.

Abbreviations: ALT, alanine transferase; AST, aspartate transferase; BMI, body mass index; CI, confidence interval; DBP, diastolic blood pressure; eGFR, estimated glomerular filtration rate; GGT, gamma‐glutamyl transferase; HDL‐C, high‐density lipoprotein cholesterol; LDL‐C, low‐density lipoprotein cholesterol; OR, odds ratio; RC, remnant cholesterol; SBP, systolic blood pressure; TG, triglyceride; WHR, waist‐to‐hip ratio.

### 3.4 Stratification analysis

The results of the stratification analysis were presented in Table 4. RC was significantly associated with diabetes regardless of whether participants were overweight or not (BMI <24 Kg/m^2^: Q3: OR: 1.148, 95% CI: 1.015–1.297, *p* = .028; Q4: OR: 1.175, 95% CI: 1.013–1.364, *p* = .033; BMI ≥24 Kg/m^2^: Q3: OR: 1.124, 95% CI: 1.016–1.243, *p* = .024; Q4: OR: 1.125, 95% CI: 1.006–1.259, *p* = .033). RC and diabetes were significantly correlated when SBP <140 mm Hg and DBP <90 mm Hg (Q3: OR: 1.126, 95% CI: 1.010–1.255, *p* = .032; Q4: OR: 1.179, 95% CI: 1.039–1.338, *p* = .011), but there was no correlation when SBP ≥140 mm Hg or DBP ≥90 mm Hg. For participants <55 years of age, RC was significantly associated with diabetes (Q3: OR: 1.238, 95% CI: 1.047–1.427, *p* = .003; Q4: OR: 1.322, 95% CI: 1.124–1.555, *p* = .001). However, there was no association between RC and diabetes among participants aged 55–65 years or ≥65 years. When 60 ≤ eGFR ≤90 ml/min per 1.73 m^2^, RC was significantly related to diabetes (Q3: OR: 1.170, 95% CI: 1.044–1.311, *p* = .007; Q4: OR: 1.211, 95% CI: 1.067–1.374, *p* = .003), whereas RC and diabetes were not associated when eGFR ≥90 ml/min per 1.73 m^2^ or eGFR <60 ml/min per 1.73 m^2^.

**TABLE 4:** Stratification analysis

Variable	Group 1	Group 2		Group 3		Group 4	
	Reference	OR (95% CI)	*p* value	OR (95% CI)	*p* value	OR (95% CI)	*p* value
BMI, Kg/m^2^							
BMI < 24	1	1.088 (0.968, 1.224)	.158	1.148 (1.015, 1.297)	.028	1.175 (1.013, 1.364)	.033
BMI ≥ 24	1	1.047 (0.945, 1.159)	.38	1.124 (1.016, 1.243)	.024	1.125 (1.006, 1.259)	.039
Blood pressure, mm Hg							
SBP < 140 and DBP < 90	1	1.072 (0.964, 1.192)	.202	1.126 (1.010, 1.255)	.032	1.179 (1.039, 1.338)	.011
SBP ≥ 140 or DBP ≥ 90	1	1.035 (0.925, 1.157)	.551	1.100 (0.984, 1.230)	.092	1.085 (0.957, 1.230)	.205
Age, years old							
age < 55	1	1.095 (0.952, 1.258)	.204	1.238 (1.047, 1.427)	.003	1.322 (1.124, 1.555)	.001
55 ≤ age < 65	1	1.028 (0.912, 1.159)	.648	1.114 (0.989, 1.255)	.075	1.108 (0.967, 1.270)	.138
age ≥ 65	1	1.072 (0.924, 1.243)	.361	1.035 (0.890, 1.203)	.658	1.040 (0.871, 1.241)	.667
eGFR, ml/min per 1.73 m^2^							
eGFR ≥ 90	1	1.033 (0.929, 1.148)	.549	1.107 (0.991, 1.236)	.073	1.053 (0.922, 1.203)	.443
60 ≤ eGFR ≤ 90	1	1.118 (0.995, 1.255)	.06	1.170 (1.044, 1.311)	.007	1.211 (1.067, 1.374)	.003
eGFR <60	1	0.883 (0.541, 1.441)	.618	0.968 (0.609, 1.539)	.89	1.508 (0.911, 2.495)	.11

*Note*: Adjusted for age, sex, center, myocardial infarction, stroke, coronary heart disease, occupation, smoking habit, drinking habit, physical activity level, family history of diabetes, ALT, AST, GGT, eGFR, SBP, DBP, BMI, WHR, insulin, HDL‐C, LDL‐C, and TG.

Abbreviations: ALT, alanine transferase; AST, aspartate transferase; BMI, body mass index; CI, confidence interval; DBP, diastolic blood pressure; eGFR, estimated glomerular filtration rate; GGT, gamma‐glutamyl transferase; HDL‐C, high‐density lipoprotein cholesterol; LDL‐C, low‐density lipoprotein cholesterol; OR, odds ratio; RC, remnant cholesterol; SBP, systolic blood pressure; TG, triglyceride; WHR, waist‐to‐hip ratio.

## 4 DISCUSSION

### 4.1 Main findings

The present study's findings suggested a significant association between RC and diabetes in the general Chinese population. In addition, RC was still associated with diabetes even when TG, LDL‐C, or HDL‐C was at the appropriate level recommended by guidelines. Moreover, people with elevated RC were more likely to develop diabetes, especially females, those with normal blood pressure, those with an eGFR between 60 and 90 ml/min per 1.73 m^2^, and those younger than 55 years of age. Hence, people with these characteristics should pay more attention to monitoring RC values and timely correction of risk factors to prevent the occurrence of diabetes. To the best of our knowledge, this is the first large sample, multicenter study to investigate the relationship between RC and diabetes in the general population.

### 4.2 Previous studies

Studies on the relationship between RC and diabetes are scarce and have some minor limitations. A single‐center study of 15 464 Japanese found that RC could predict diabetes.^19^ However, their study did not measure PBG, so their definition of diabetes did not include PBG≥11.1, the definition of diabetes in the current multicenter study strictly followed the American Diabetes Association's criteria, making it more accurate and scientific, and the current study was multicenter and fully adjusted for confounders. Szili‐Torok et al^20^ analyzed 480 patients who had undergone kidney transplantation, and they found a significant association between RC and new‐onset diabetes after kidney transplantation. Zhou et al^21^ found a positive correlation between RC and diabetes in 13 721 Chinese hypertensive patients. Nevertheless, the current study population was the large sample general Chinese population, including subjects with various chronic diseases and healthy subjects; hence, the findings of the current study were more generally applicable.

### 4.3 Potential mechanisms

The reason for the association between RC and diabetes remains unclear. Atherosclerosis might be a major contributor. Numerous investigations have shown a strong link between atherosclerosis and hyperglycemia.^22^, ^23^, ^24^ Elevated RC levels aided in the atherosclerosis progression,^10^, ^25^, ^26^, ^27^ which causes hyperglycemia. RC could be penetrable to the arterial wall, be phagocytosed by macrophages, and result in the formation of foam cells. Furthermore, RC speeds up the creation of foam cells via upregulating scavenger receptor expressions.^26^ Atherosclerosis lowers blood circulation to the pancreas and impairs pancreatic function, which reduces insulin secretion levels and causes hyperglycemia.^28^ Atherosclerosis also causes liver dysfunction and reduces glycogen production in the liver, thus leading to hyperglycemia.^29^

Insulin resistance (IR) could potentially account for the link between RC and diabetes. In a study of 86 nonobese Japanese diabetics, researchers found that the IR group had higher levels of RC than the insulin‐sensitive group.^30^ Funada et al discovered that RC could forecast IR independently.^31^ A recent study found that empagliflozin reduced RC in diabetic patients, which was highly related to a reduction in IR.^32^ Besides, RC could directly disrupt pancreas islet β‐cells' function, resulting in decreased insulin secretion.^33^

### 4.4 The association between RC and diabetes when TG, LDL‐C, or HDL‐C is within the appropriate range recommended by the guidelines

“Atherosclerotic lipid triad,” characterized by elevated levels of TG and LDL‐C and reduced levels of HDL‐C, is thought to predict diabetes risk.^19^ Nevertheless, in the current study, when TG was <1.7 mmol/L, LDL‐C <3.4 mmol/L, or HDL‐C ≥1.0 mmol/L, the association between RC and diabetes remained significant. Because many human cells could degrade TG, but none could degrade cholesterol, the content of cholesterol in the residue (RC) is the primary cause of atherosclerosis.^27^ An Icelandic study has shown that the detrimental effects of a TG‐elevating gene variant are mediated by atherogenic effects of the cholesterol component of RC.^34^ On the other hand, increased RC concentrations have been reported to lead to atherosclerosis risk even in individuals with normal TG levels.^35^ As our results show, even if TG levels are appropriate, elevated RC and the corresponding risk of diabetes could not be ignored.

Varbo et al found that elevated LDL‐C could generate atherosclerosis, but not inflammation, and the inflammatory component of atherosclerosis was driven by elevated RC.^27^ Despite lowering LDL‐C to suggested levels, there is a significant adverse residual risk of atherosclerosis,^36^ which could be explained by the link between RC and inflammation. In addition, because RC is ingested in an uncontrolled way through scavenger receptors, RC causes greater damage to *β* cells than LDL‐C.^20^

It has been demonstrated that there is no causality between low HDL‐C and atherosclerotic incidents, and low HDL‐C is merely a strong symbol of elevated RC.^37^ After getting into the intima, HDL particles could penetrate the media and leave the arterial wall,^13^, ^38^ but RC might be trapped in the intima and generate the foam cells, causing regional injury and inflammation.^13^, ^38^ Moreover, TGRL promotes the remodeling of HDL‐C into smaller, cholesterol‐poor particles that might lack atherosclerotic protection regardless of HDL‐C levels.^39^ Combined with the results of the current study, we recommend that people whose TG, LDL‐C, or HDL‐C levels is at the appropriate level recommended by the guidelines should also be vigilant against the increased risk of atherosclerosis and diabetes associated with elevated RC.

### 4.5 Gender difference

The gender difference in older populations might be due in part to the gender‐specific hormones. Our participants' median age (Q1–Q3) was 57 (52, 64), indicating that the women in the current study were either perimenopausal or postmenopausal. The atherogenic effect of RC is more pronounced in women at this stage.^40^ Ossewaarde et al observed a 15% reduction in RC levels in a group of healthy Dutch postmenopausal women given estrogen + progestin combination therapy for 3 months.^41^ Hence, perimenopausal and postmenopausal women are more prone to develop RC abnormalities owing to hormonal changes, increasing their risk of atherosclerosis and diabetes.

### 4.6 Stratification analysis

Previous studies demonstrated that the causality between RC and low‐level inflammation still existed even in participants who were neither overweight or obese,^27^ suggesting that the relationship between elevated RC and low‐grade inflammation is not attributable to weight gain. The present study also found that RC and diabetes remained strongly associated regardless of whether the participant was overweight or not. Zhou et al^21^ found a positive correlation between RC and diabetes in 13 721 hypertensive patients; however, RC and diabetes were associated only in persons with normal blood pressure in the current investigation. We speculate that this is because the number of hypertension patients in the current research was modest in comparison to Zhou's team's investigation. Further validation of the relationship between RC and diabetes in a larger sample of hypertensive patients is needed in the future. We found the correlation was just significant in participants younger than 55 years old, not in older participants. We hypothesize that older adults tend to maintain healthy lifestyle habits and possess better compliance, which might help to a prevention of dyslipidemia and diabetes. Prior literature has demonstrated a U‐type relation between eGFR and all‐cause mortality,^42^ highlighting the significance of both high and low eGFR. The current study found a correlation between RC and diabetes only in participants with 60 ≤ eGFR ≤90 ml/min per 1.73 m^2^. We consider that the damage caused by low or high eGFR might cover up the pathogenicity of elevated RC.

### 4.7 Limitations

The current study benefited from a vast collection of multiple community‐based samples, broadly representative of most Chinese people. Meanwhile, the current study also comprehensively adjusted for major risk factors and conducted a detailed stratification analysis. However, some limitations still remained. First, VLDL residues are a major component of RC during fasting, so the role of chylomicron residues may be underestimated. Second, the current study was cross‐sectional, so we could we could make only correlational inferences, not causal ones.

## 5 CONCLUSIONS

The present study observed that elevated RC is significantly associated with diabetes in the Chinese general population, even when TG, LDL‐C, or HDL‐C was at the appropriate level recommended by guidelines. Population with elevated RC are at higher danger of diabetes, especially in subjects with normal blood pressure, 60 ≤ eGFR ≤ 90 ml/min per 1.73 m^2^, younger than 55 years old, and female. The current study's findings expand RC's application and provide new approaches to preventing diabetes. Calculating RC is an affordable method. It is necessary to measure RC along with monitoring other lipid markers in general people, even if traditional lipid components are at appropriate levels.

## AUTHOR CONTRIBUTIONS

All authors have read and approved the final manuscript. Binqi Li contributed to the conception and design of the study. Binqi Li, Xin Zhou, Weiqing Wang, Zhengnan Gao, Li Yan, Guijun Qin, Xulei Tang, Qin Wan, Lulu Chen, Zuojie Luo, Guang Ning, and Yiming Mu recruited the subjects and supervised the study. Binqi Li analyzed the data and wrote the initial draft of the paper. Yiming Mu and Binqi Li contributed to the manuscript's writing, reviewing, and revising.

## FUNDING INFORMATION

The study is supported by Beijing Municipal Science and Technology Commission Project (Z201100005520014), the Chinese Society of Endocrinology, the Key Laboratory for Endocrine and Metabolic Diseases of Ministry of Health (1994DP131044), the National Key New Drug Creation and Manufacturing Program of Ministry of Science and Technology (2012ZX09303006‐001), the National High Technology Research and Development Program of China (863 Program, 2011AA020107), National Science Foundation of China (81300717), National Science and Technology Major Project 288 (2011ZX09307‐001‐08).

## CONFLICT OF INTEREST

The authors declare no competing interests.

## Data Availability

The data sets are not freely available due to protection of participants' privacy.
